# Breaking the spatial reciprocity with Janus metamaterials

**DOI:** 10.1038/s41377-019-0175-5

**Published:** 2019-07-10

**Authors:** Lingling Huang

**Affiliations:** Key Laboratory of Photoelectronic Imaging Technology and System, Ministry of Education, School of Optics and Photonics, Beijing Institute of Technology, 100081 Beijing, China

**Keywords:** Metamaterials, Optical data storage

## Abstract

Switching of the observation direction as well as the polarization channels of chiral Janus metamaterials may result in different reconstructed images.

Chirality is a symmetry property of an object. A chiral object cannot be superposed with its mirror image through simple rotation or translation. The object and its mirror image are called enantiomers^1^. Chiral objects can be found in our daily life, ranging from amino acids, nucleic acids, proteins, and screws to our hands and feet. Special optical properties arise from chiral structures. For example, right- or left-handed circularly polarized (RCP or LCP) light can interact with chiral objects in different ways and exhibit distinct physical phenomena, such as circular dichroism and circular birefringence, jointly referred to as optical activity. These two important phenomena arise from the fact that chiral objects possess different effective refraction indices and absorptions under RCP and LCP light, which result in different phase delays and polarization conversions. However, the chiral optical responses of most biomolecules or other chiral objects in nature are generally very weak. To overcome this problem, various chiral metamaterials have been proposed to realize strong light-matter interactions to achieve giant optical activity, a negative refractive index and so on^2,3^.

Advances in metamaterials/metasurfaces have led to many breakthroughs for tailoring light, such as for beam shaping, holography and information processing^4,5^. With the aim of reducing ohmic loss and solving nanofabrication challenges for bulk metamaterials, planar metasurfaces have attracted intense interest over the past decade. By delicately designing the geometries and rotations of the unit cell, together with smart patterning, various intriguing phenomena have been observed. Resonance phase and geometric phase effects have been identified, and various light modulation mechanisms, such as phase, amplitude, polarization, angular momentum and frequency modulation, have been revealed based on metasurfaces^6^. Among these, spin-controlled wavefront shaping with geometric metasurfaces can exhibit dual functionality when the helicity of the incident circularly polarized light changes. However, due to the lack of spatial symmetry along the propagation directions, the planar metasurfaces reported thus far have exhibited reciprocal behavior for opposite direction observations. Hence, the study of metamaterials/metasurfaces for direction-control-related phenomena is particularly appealing.

Recently, Chen et al.^7^ reported direction-controlled polarization-encrypted data storage based on 3D plasmonic helical nanoapertures. The helical nanoapertures can be fabricated using a one-step grayscale focused ion beam milling method. In the forward direction, circular dichroism is achieved in the experiment due to the spin-dependent mode coupling process inside the helical nanoaperture, and most of the incident circularly polarized light is converted into linearly polarized light (Fig. 1). In the backward direction, the circular dichroism disappears, but the plasmonic helical nanoapertures exhibit giant linear dichroism. By utilizing these novel properties, the authors designed a ‘Janus’ metamaterial composed of the two enantiomers of helical nanoapertures with specific orientation angles to achieve bidirectional imaging. Under a certain helicity of circularly polarized light, a binary QR code image can be obtained in the forward direction. In contrast, under illumination with linearly polarized light from the backward direction to utilize the linear dichroism, a distinct grayscale image is generated based on Malus’s law. Such Janus metamaterials extend the functionality by encoding the observation direction as a readout key and address the issues of both circular and linear dichroism.

Based on the opportunity to precisely control the optical properties with metamaterials/metasurfaces, this work can further boost nonreciprocal wavefront engineering applications, that is, to break the spatial or time reversal symmetry of metadevices. For example, asymmetric transmission phenomena based on different polarization bases, together with full phase and amplitude modulation capabilities, can greatly expand the degree of freedoms for controlling light in different directions in nanoscale optical devices^8^. We can envision that such methods may pave the way toward integrated photonic devices, multifunctional polarization control, data encryption and decryption, and optical information processing.

**Fig. 1:**
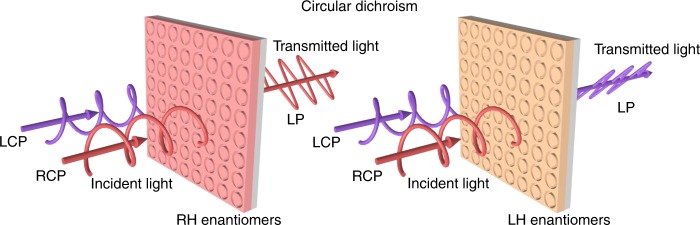
Circular dichroism of a Janus metasurface. For metasurfaces composed of arrays of RH enantiomers, the incident RCP light can be converted into linearly polarized light. However, the transmission of incident LCP light is very low. The results are reversed for the metasurfaces composed of arrays of LH enantiomers
